# Prognostic models for immunotherapy in non-small cell lung cancer: A comprehensive review

**DOI:** 10.1016/j.heliyon.2024.e29840

**Published:** 2024-04-17

**Authors:** Siqi Ni, Qi Liang, Xingyu Jiang, Yinping Ge, Yali Jiang, Lingxiang Liu

**Affiliations:** a Department of Oncology, The First Affiliated Hospital of Nanjing Medical University, Nanjing 210029, China; b The Friendship Hospital of Ili Kazakh Autonomous Prefecture Ili & Jiangsu Joint Institute of Health, Yining 835000, Xinjiang Uygur Autonomous Regio, China

**Keywords:** Prognostic model, Immune checkpoint inhibitors, Non-small cell lung cancer

## Abstract

The introduction of immune checkpoint inhibitors (ICIs) has revolutionized the treatment of lung cancer. Given the limited clinical benefits of immunotherapy in patients with non-small cell lung cancer (NSCLC), various predictors have been shown to significantly influence prognosis. However, no single predictor is adequate to forecast patients' survival benefit. Therefore, it's imperative to develop a prognostic model that integrates multiple predictors. This model would be instrumental in identifying patients who might benefit from ICIs. Retrospective analysis and small case series have demonstrated the potential role of these models in prognostic prediction, though further prospective investigation is required to evaluate more rigorously their application in these contexts. This article presents and summarizes the latest research advancements on immunotherapy prognostic models for NSCLC from multiple omics perspectives and discuss emerging strategies being developed to enhance the domain.

## 1 Introduction

**Fig. 1:**
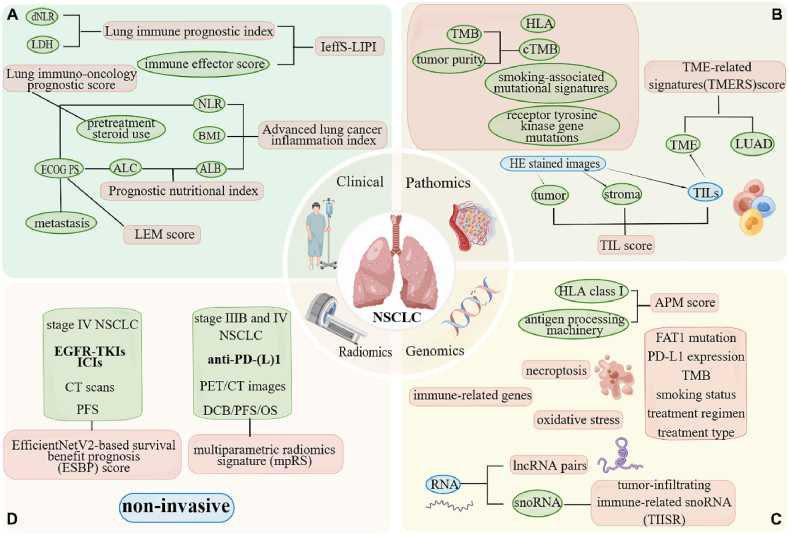
Prognostic models for immunotherapy in NSCLC by Figdraw (www.figdraw.com). (A) Clinical-based prognostic models. (B) Pathomics-based models. (C) Genomics-based models. (D) Radiomics-based models.

Lung cancer accounts for 21 % of all cancer-related deaths and is the leading cause of cancer death worldwide [1]. Targeting immune checkpoint pathways has initiated the use of immune checkpoint inhibitors (ICIs), which have seen remarkable clinical advancements in the past decade, especially concerning non-small cell lung cancer (NSCLC), bladder cancer and melanoma [2]. However, only a small fraction(10–30 %) of patients with solid tumors respond to immunotherapy [3]. Therefore, pinpointing patients who will benefit from immunotherapy has become a paramount challenge. At present, a plethora of biomarkers have been proposed to identify NSCLC patients receptive to ICIs. These biomarkers include tumor mutation burden (TMB), programmed death ligand 1 (PD-L1) expression, and microsatellite mismatch repair [[4], [5], [6]]. Yet, these biomarkers come with their own set of challenges in clinical practice. These challenges range from varying cut-off values, inconsistencies across detection platforms, to a limited use of sequencing [7]. Since a single predictor often falls short, it's crucial to develop prognostic models that amalgamate multiple predictors. Such models can more effectively pinpoint patients poised to benefit from ICIs. This paper introduces and delves into these models (Fig. 1 and Table 1), providing a comprehensive overview of their potential and applications.

**Table 1:** Characteristics of prognostic models for immunotherapy in NSCLC.

First Author	Year	Cancer Type	Omics	ICIs Type	Study Design	Number		Outcome		Method	Type of Model	Discrimination Performance	
						Development	Validation	Primary	Secondary			Development	Validation
Mezquita [8]	2018	NSCLC	clinical	aPD1/PDL1	retro	161	305	OS	PFS/DCR	Cox PH	PI	–	
Sorich [9]	2019	NSCLC	clinical	aPDL1 (atezolizumab)	randomised trials	161	306	OS	PFS/ORR	Cox PH	PI	0.63	0.61
Wang [10]	2020	NSCLC	clinical	aPD1/PDL1	retro	216	114	PFS	OS	Cox PH	PI	–	
Mazzaschi [11]	2020	NSCLC	clinical	aPD1/PDL1	prospective	109		PFS	OS	Cox PH	risk score	0.9	
Shoji [12]	2019	NSCLC	clinical	aPD1/PDL1	retro	102		PFS	OS	Cox PH	PI	0.694	
Mountzios [13]	2021	aNSCLC	clinical	aPDL1	retro	672	444	OS	PFS	Cox PH	PI	0.6893	
Li [14]	2021	NSCLC	clinical	aPD1/PDL1	retro	87	171	PFS	DCB/NDB/ORR/OS	Cox PH	risk score	–	
Banna [15]	2021	aNSCLC	clinical	aPDL1	retro	201	583	OS	–	Cox PH	risk score	0.63	
Zeng [16]	2021	NSCLC	clinical	aPD1/PDL1	retro	86	44	PFS	–	Cox PH	nomogram	0.725	0.688
Dimitrakopoulos [17]	2022	aNSCLC	clinical	aPD1	retro	112	626	PFS	OS	Cox PH	risk score	–	0.605
Johannet [18]	2020	pan-cancer	clinical	aPD1/PDL1/CTLA4	retro	629		PFS	OS	Cox PH	PI	–	
Perrone [19]	2023	advanced cancer	clinical	aPD1/PDL1/CTLA4	retro	68	–	OS	PFS/CB	Cox PH	biomarker	–	
Unger [20]	2023	advanced LUSC	clinical	aPD1/CTLA4	retro	158	–	OS	PFS	Cox PH	risk score	–	
Hopkins [21]	2020	aNSCLC	clinical	aPDL1 (atezolizumab)	randomised trials	751	797	OS	PFS	Cox PH	risk score	0.72	0.76
Anagnostou [22]	2020	NSCLC	pathomics	aPD1/PDL1/CTLA4	retro	89	34	PFS	OS	Cox PH	risk score	–	
Huang [23]	2022	LUAD	pathomics	aPD1/PDL1/CTLA4	TCGA	479	1151	OS	–	Cox PH	risk score	1-year OS 0.720 3-year OS 0.679 5-year OS 0.628	
Yu [24]	2019	aNSCLC	pathomics	aPD1/PDL1/CTLA4	retro	14395	–	OS	PFS/ORR	Cox PH	combination	3-year OS 0.659 5-year OS 0.665	–
Ghiringhelli [25]	2023	NSCLC	pathomics	aPD1/PDL1	retro	133	132	PFS	OS	Cox PH	combination	–	
Peng [26]	2023	NSCLC	pathomics	aPD1/PDL1/CTLA4	TCGA	344	148	OS	–	Cox PH	risk score	1-year OS 0.662 3-year OS 0.601 5-year OS 0.671	
Rakaee [27]	2023	NSCLC	pathomics	aPD1/PDL1	retro	446	239	PFS	OS	Cox PH	risk score	0.77	
Thompson [28]	2020	NSCLC	genomics	aPD1/PDL1	retro	51	–	PFS	OS	Cox PH	risk score	PFS 0.84 OS 0.70	–
Zhu [29]	2021	NSCLC	genomics	aPD1/PDL1/CTLA4	retro	158	121	PFS	–	Cox PH	nomogram	6-month PFS 0.763 12-month PFS 0.871	
Yi [30]	2021	LUAD	genomics	aPD1/PDL1	TCGA	331	166	OS	–	Cox PH	risk score	3-year OS 0.74 5-year OS 0.70	3-year OS 0.69 5-year OS 0.64
Huang [31]	2022	LUAD	genomics	aPD1/PDL1/CTLA4	TCGA	296	149	OS	–	Cox PH	risk score	1-year OS 0.59 3-year OS 0.69 5-year OS 0.74	
Zhang [32]	2022	LUAD	genomics	aPD1/CTLA4	TCGA	594		OS	–	Cox PH	risk score	0.826	
Liu [33]	2021	LUAD	genomics	aPD1/PDL1/CTLA4	TCGA	232	232	OS	–	Cox PH	risk score	0.769	
Wan [34]	2021	LUAD	genomics	aPD1/PDL1/CTLA4	TCGA	479	–	OS	–	Cox PH	risk score	3-year OS 0.83 5-year OS 0.82	–
Li [35]	2022	NSCLC	genomics	aPDL1	TCGA	999	570	OS	–	Cox PH	risk score	0.700	
Smith [36]	2023	NSCLC	genomics	aPD1/PDL1/CTLA4	retro	298	226	OS	PFS	Cox PH	risk score	0.628	0.595
Zeng [37]	2023	LUAD	genomics	aPD1/PDL1/CTLA4	TCGA	495	–	OS	–	Cox PH	risk score	0.7	
Xie [38]	2023	NSCLC	genomics	aPD1/PDL1/CTLA4	TCGA	1149	–	OS	–	Cox PH	risk score	1-year OS 0.604 3-year OS 0.610 5-year OS 0.550	–
Deng [39]	2022	aNSCLC	radiomics	aPD1/PDL1/CTLA4	retro	–	129	PFS	–	Cox PH	risk score	–	–
Ventura [40]	2023	aNSCLC	radiomics	aPD-1 (Pembrolizumab)	retro	44	–	PFS	–	Cox PH	radiomics signature	PET-skewness 0.69 PET-median 0.75	
Humbert [41]	2022	aNSCLC	radiomics	aPD1/PDL1	prospective	92	45	PFS	OS	Cox PH	radiomics signature	2-year OS 0.727	2-year OS 0.707 3-year OS 0.782
Mu [42]	2020	aNSCLC	radiomics	aPD1/PDL1/CTLA4	retro	194	47	PFS	OS	Cox PH	radiomics signature	0.86	0.83
							48						0.81
Mu [43]	2021	aNSCLC	radiomics	aPD1/PDL1/CTLA4	retro	123	52	PFS	OS	Cox PH	radiomics signature	0.77	0.75
							35						0.74

aNSCLC, advanced NSCLC; aPD1/PDL1/CTLA4, anti-PD-1/PD-L1/CTLA-4; retro, retrospective; Cox PH, cox proportional hazard analysis; PI, prognostic index.

## 2 Clinical-based prognostic models

### 2.1 Lung immune prognostic index (LIPI)

In 2018, Mezquita et al. firstly introduced the pre-treatment LIPI for patients with advanced NSCLC undergoing programmed death 1 (PD-1)/PD-L1 inhibitor therapy. LIPI comprises two metrics: the derived neutrophil-to-lymphocyte ratio (dNLR) and lactate dehydrogenase (LDH) levels (Fig. 1A). Using a dNLR threshold of 3 and an LDH level exceeding the normal upper limit, patients were categorized into three LIPI groups: good (0 factor), moderate (1 factor), and poor (2 factors). The respective median overall survival (OS) for these groups spanned 3, 10, and 34 months, while the median progression-free survival (PFS) were 2.0, 3.7, and 6.3 months (*P* < 0.001). However, no discernible difference was noted across varied LIPI groups in chemotherapy-only treatments [8]. Contrarily, a pooled analysis by Sorich from clinical trials incicated LIPI's association with both survival (*P* < 0.001) and response (*P* = 0.005) in NSCLC patients administered docetaxel. Similarly, LIPI exhibited significant correlations with OS (*P* < 0.001), PFS (*P* < 0.001), and response (*P* < 0.001) in patients treated with atezolizumab [44]. Xu et al. posited that the disparity in LIPI's predictive capability for chemotherapy outcomes stemmed from Sorich's predominantly Caucasian cohort [45]. Responding, Sorich et al. argued that the patient group in Mezquita's report was predominantly Caucasian. They further emphasized LIPI's correlation with the survival of 128 Asian patients on docetaxel, revealing comparable predictive associations for both Asian and Caucasian patients (*P* = 0.003) [9]. Both parties concurred that integrating LIPI with an immune-related molecular signature would refine the precision medicine framework.

In a 2019 analysis of 11 randomized trials focused on metastatic NSCLC, Kazandjian and colleagues emphasized the prognostic significance of baseline LIPI, irrespective of treatment modalities-be it immunotherapy, chemotherapy, or targeted therapy [46]. Long et al. proposed alternative cutoff values, an in-depth delineation of lymphocyte subpopulations coupled with a consideration of metastatic lesion locations [47,48]. A separate study revealed that an improved LIPI during the second treatment cycle was indicative of better PFS for patients on mono-immunotherapy, but this was not observed in those receiving a combination of ICIs and chemotherapy [10]. This underscores the potential value of dynamic LIPI monitoring for timely prognosis assessment in immunotherapy recipients.

In a 2020 study, Mazzaschi et al. undertook prospective collection of baseline peripheral blood from 109 NSCLC patients undergoing ICIs treatment. They constructed an immune effector score (I_eff_S) rooted in a composite risk model reflecting tumor-host interactions. The IeffS risk factors encompassed elevated soluble PD-L1, diminished CD8^+^PD-1^+^, and scarcity of NK cells, pointing towards reduced ICIs benefits (*P* < 0.01). Notably, the amalgamation of I_eff_S with LIPI markedly influenced survival outcomes (PFS, HR = 4.61; OS, HR = 4.03) and ICIs responsiveness (*P* < 0.001) [11].

While ICIs exhibit commendable efficacy with sustained responses, their overall response rate in NSCLC patients remains subdued. Clinical prediction models, delineate patient subgroups with varied prognoses through clinicopathological data. These models provide reasonable and personalized vital tools to facilitate clinical decisions [49].

### 2.2 Advanced lung cancer inflammation index (ALI)

In 2013, Jafri et al. firstly proposed the ALI (Fig. 1A) as a prognostic indicator for metastatic NSCLC. It amalgamates three parameters: body mass index (BMI), ALB, and neutrophil-lymphocyte ratio (NLR), collectively mirroring the host's systemic inflammation [50]. A 2021 extensive multicenter retrospective analysis revealed a robust association between a high ALI (>18) an enhanced OS (*P* < 0.001) in mono-immunotherapy recipients. However, this correlation was absent in those undergoing combined immunotherapy and chemotherapy (*P* = 0.111). Notably, for PD-L1 high expressers, an elevated ALI (>18) could potentially obviate the need for additional chemotherapy [13].

### 2.3 LEM score

In 2021, Li et al. forulated a risk scoring system encapsulating ALC (L), the Eastern Cooperative Oncology Group performance status (ECOG PS, E), and lung/pleural metastasis (M). A surging LEM score signals a diminished response and reduced PFS. Additionlly, they discerned that patients with epidermal growth factor receptor (EGFR) mutations registered elevated LEM scores compared to their wild-type counterparts, thereby implying a compromised response to ICIs [14].

### 2.4 Lung immuno-oncology prognostic score (LIPS)

In 2021, Banna et al. delved into the prognostic determinants for advanced NSCLC patients exhibiting PD-L1 levels of ≥50 % and receiving first-line immunotherapy. They introduced the LIPS-3 model, including ECOG PS 2, NLR<4 and pre-treatment steroid usage. Based on the accumulated risk factors, patients were segmented into three risk categories, boasting one-year OS rates of 78.2 %, 53.8 %, and 10.7 %. The Harrell C index for this model stood at 0.65 in the training set (n = 201) and 0.66 in the validation cohort (n = 583) [15].

### 2.5 Patras immunotherapy score (PIOS)

In 2020, Dimitrakopoulos and team constructed a scoring system derived from four baseline parameters: performance status (PS) × body mass index (BMI)/lines of treatment (LOT) × age. This was formulated after analyzing 112 advanced NSCLC patients treated with anti-PD-1 monotherapy, either nivolumab or pembrolizumab [51]. By 2022, Dimitrakopoulos and colleagues validated the clinical application of PIOS using an external cohort (n = 626). Notably, individuals with elevated PIOS scores manifested enhanced PFS and OS across both univariate and multivariate evaluations (AUC = 0.605) [17].

### 2.6 Prognostic nutritional index (PNI)

In 1984, Onodera et al. identified PNI as a prognostic beacon across various malignant tumors [[52], [53], [54]]. This index, derived from the absolute lymphocyte count (ALC) and albumin (ALB), serves as a measure of the patient's immunotrophic status [55]. In a 2019 retrospective study, Shoji et al. revealed that NSCLC patients with lower pre-treatment PNI values tended to exhibit poor ICIs response [12]. Earlier studies indicated that patients with a BMI ≥25 experienced superior clinical results with anti-PD-1/PD-L1 therapies compared to those having a BMI <25 [56]. Johannet's 2020 study on 629 advanced cancer patients affirmed that diminishing nutritional health prior to immunotherapy, rather than static BMI values, negatively swayed the ICI response, impacting both PFS (*P* = 0.02) and OS (*P* < 0.001) [18].

### 2.7 Other multivariate clinical models

The symptom burden in advanced NSCLC is considerable, with manifestations like cough, shortness of breath, and chest pain severely impinging on patients' functional capacity and quality of life (QOL) [57,58]. Concrete evidence delineating symptom progression is paramount for informed therapeutic decisions. In 2023, Joseph et al. retrospectively analyzed 158 patients from LungMAP-S1400I and deduced that the combined regimen of nivolumab and ipilimumab didn't outshine the efficacy of nivolumab as a monotherapy. Intriguingly, a preliminary baseline risk model encompassing appetite loss and breathlessness recognized patients facing an over three-fold escalation in progression risk (HR = 3.06, 95 % CI, 1.88–4.98, *P* < 0.001). Furthermore, a model integrating work limitations and appetite loss identified individuals with a staggering five-fold surge in mortality risk (HR = 5.60, 95 % CI, 3.27–9.57, *P* < 0.001) [20].

Cholesterol metabolism has emerged a promising and appealing biomarker [59]. The intricate biology and therapeutic strategies for metastatic renal cell carcinoma (mRCC) and NSCLC appear intertwined with cholesterol efflux mechanisms, especially those mediated by serum transporters, ABCA1 and ABCG1, as well as passive diffusion [60]. In 2023, Perrone et al. retrospectively assessed mRCC and advanced NSCLC patients undergoing ICIs treatments (n = 70). Their findings underscored the favorable correlation of passive diffusion with OS, PFS, and clinical advantage. However, they advocated for further prospective research to confirm the findings [19].

In 2021, Zeng et al. retrospectively analyzed 130 patients with stage IIIA-IVB NSCLC receiving immunotherapy combined with chemotherapy, and developed a PFS nomogram based on 4 pivotal factors: bone metastasis, dNLR, smoking status, and PD-L1 status. Intriguingly, the low-risk patient group exhibited an elevated median PFS (mPFS) (*P* < 0.001). In terms of accuracy, this model's C-index stood at 0.725 for the training cohort and 0.688 for the validation set [16].

In 2020, Hopkins et al. developed and validated a prognostic tool to identify beneficiary patients with advanced NSCLC receiving atezolizumab based on large clinical trials. This tool integrates multiple parameters: PD-L1 expression, dNLR, C-reactive protein (CRP), LDH, ALB, performance status, the elapsed time post-metastasis diagnosis, and the tally of metastatic sites. The low-risk group benefit most from atezolizumab. Notably, this research marked the inaugural revelation of CRP as a potent predictor of OS for advanced NSCLC patients undergoing atezolizumab treatment. A diminished CRP level was robustly linked with extended OS (*P* < 0.001, *c* = 0.66) [21].

### 2.8 Pathomics-based models

While TMB has been closely associated with immunotherapy responses across multiple cancers [61], Anagnostou et al. posited that tumors with low-purity might skew TMB evaluations in 2020. To refine the prediction accuracy for ICIs efficacy, they introduced a corrected TMB (cTMB) that accounts for tumor purity. This enhanced predictive model encompassed cTMB, receptor tyrosine kinase gene mutations, smoking-linked mutational signatures, and human leukocyte antigen (HLA). Notably, patients exhibiting higher risk scores presented with a notably reduced OS (*P* = 0.0001) [22].

Previous researches have underscored the potential of various TME biomarkers in prognostic evaluations and gauging ICIs responsivenes [62,63]. In 2022, Huang et al. crafted a TME-centric prognostic classification model (Fig. 1B) tailored for lung adenocarcinoma (LUAD) patients (n = 479). Elevated tumor microenvironment-related signatures (TMERSscore) indicated a grim prognosis and showcased a robust association with tumor malignancy. Furthermore, a diminished TMERSscore forecasted a favorable ICIs response, potentially offering incremental predictive prowess over prevailing biomarkers [23].

In 2023, Rakaee et al. undertook a retrospective analysis of 685 NSCLC patients on ICIs, and developed a machine learning-driven tumor-infiltrating lymphocyte (TIL) scoring system. This method autonomously quantifies tumor, stroma, and TIL cells within hematoxylin-eosin stained samples. Irrespective of the treatment regimen, elevated TIL levels correlated with enhanced response rates, improved PFS, and OS. Combinatorial metrics like TIL/PD-L1 and TMB/PD-L1 outperformed solitary PD-L1 in discerning immunotherapy beneficiaries. Notably, TIL surpassed TMB in predicting ICIs outcomes for PD-L1-negative patients [27].

In 2019, Yu et al. analyzed 14395 advanced NSCLC patients undergoing ICIs and concluded the optimal first-line ICIs regimen for advanced NSCLC encompassed a combination of pembrolizumab and platinum-based chemotherapy. Additionally, the presence of TMB, PD-L1 expression, and CD8^+^ T-cell tumor infiltration were correlated with favorable OS outcomes, as evidenced by a 3-year OS AUC of 0.659 and a 5-year OS AUC of 0.665 [25]. In 2023, Ghiringhelli et al. employed Immunoscore-Immune-Checkpoint (Immunoscore-IC) to analyze CD8^+^ T-cell and PD-L1^+^ cell populations (n = 206). The findings resonated with prior research that associated CD8^+^ T-cell and PD-L1^+^ cell presence with favorable ICI responses. Notably, enhanced OS and PFS outcomes showcased a strong correlation with elevated intratumoral CD8 expression [24].

While the multifaceted roles of neutrophils in the TME are recognized as pivotal in influencing cancer progression, the intricacies of cellular dynamics in tandem with NSCLC evolution remain enigmatic [64]. In 2023, Peng et al. studied 553 primary tumor samples from NSCLC patients using a multiplex immunofluorescence test. The spatial intricacies within the TME were then assessed employing the StarDist deep learning algorithm. Eventually, a robust model grounded in six genes pertinent to neutrophil differentiation was developed. This model indicated that patients with a low risk profile exhibited extended OS and potentially heightened responsiveness to immunotherapy, as reflected by a 5-year OS of 0.671 [26].

### 2.9 Genomics-based models

A profound interrelation exists between antitumor response to immunotherapy and the tumor genetics makeup of tumors. Neoantigens, borne from somatic mutations, seem to modulate immune response, bolstering the potency of ICIs against solid tumors like NSCLC [65,66]. The confluence of emerging immunotherapy and cancer genomics heralds a transformative era in the cancer care.

The CD8^+^ T cell-mediated eradication of cancer cells hinges on the adept presentation of tumor antigens via HLA class I molecules. Recently, researchers highlight that genetic erosion of the HLA class I antigen processing machinery (APM) correlates with resistance to checkpoint inhibitors [67]. In 2020, Thompson et al. formulated an APM score by harnessing 8 genes pivotal to antigen processing and presentation mechanisms: *B2M*, *CALR*, *NLRC5*, *PSMB9*, *PSME1*, *PSME3*, *RFX5*, and *HSP90AB1*. A pronounced APM score (Fig. 1C) was markedly evident in responders compared to non-responders (*P* = 0.0001). Furthermore, the receiver operating characteristic curve (AUC) for PFS and OS registered at 0.84 and 0.70, respectively [28].

Given the pivotal roles B cells assume in determining the TME and ICIs responses [68], in 2022, Li et al. spotlighted a robust risk score signature based on 23 B cell-related gene pairs (BRGPs) from 999 NSCLC samples (AUC = 0.700). Intriguingly, the high-risk cohort exhibited amplified PD-L1 expression and appeared to derive more pronounced benefits from ICIs (*P* < 0.001) [35].

Macrophages, specifically tumor-associated macrophages (TAMs), play a crucial role in the TME, influencing the trajectory of lung cancer progression [69,70]. In 2023, Xie discerned distinct disparities between M0 and M1 macrophages across various NSCLC clusters, indicating the centrality of macrophages in immunotherapy. They also explored the ramifications of macrophage-related genes (MRGs) on prognosis, noting a 3-year OS of 0.610. It also assessed their influence on the chemosensitivity of NSCLC. A pivotal function of the migration inhibitory factor (MIF) signaling pathway in NSCLC cell interactions, shedding light on novel avenues for immunotherapy [38].

In 2021, Zhu et al. pinpointed the *FAT1* mutation as a detractor in predicting sustained clinical advantages from ICIs, drawing from data across four publicly available NSCLC cohorts. Meanwhile, they unveiled a predictive model anchored on the *FAT1* mutation, boasting commendable accuracy metrics with a 6-month AUC of 0.763 and a 12-month AUC of 0.871. This model incorporated variables like PD-L1 expression, TMB, smoking patterns, treatment regimen, therapeutic categories, and the *FAT1* mutation itself [29]. In a comprehensive 2023 study, Smith and team analyzed data from 524 advanced NSCLC patients to discern the correlation between mutation profiles and therapeutic responses. Evaluating cohorts subjected to chemotherapy (n = 88), ICI (n = 226), and a chemo-ICI blend (n = 210), they employed OS-based cox-proportional hazard regression models to pinpoint mutations. This led to the formulation of mutation composite scores (MCS) tailored for each treatment type. An MCS registering a positive outcome (+1 group) was categorized as protective. A significant discovery was the superior predictive prowess of MCS (AUC = 0.628) over TMB and PD-L1 status in forecasting the prognosis of advanced NSCLC patients [36].

Immune-related genes (IRGs) modulate the onset and progression of cancer, influencing both the tumor immune microenvironment and the malignant attributes of tumor cells. Existing literature suggests that IRGs serve as prognostic indicators for several cancers, encompassing colorectal [71], cervical [72], ovarian [73] and hepatocellular carcinoma [74]. In 2021, Yi et al. delved into the LUAD cohort from the TCGA database, crafting a prognostic immune signature anchored on 17 immune-related genes. Patients categorized as low-risk showcased a more favorable prognosis [30]. In 2023, Zeng et al. utilized 495 samples from TCGA-LUAD to forge the Immune Activation Related Gene Index (IARGI). Their findings intimated that the low-risk cohort might exhibit an enhanced responsiveness to ICIs therapy, as reflected by an AUC of 0.7 [37].

Elevated levels of reactive oxygen species can catalyze tumor growth, instigating mutations and modulating cell signaling pathways [75]. In 2022, Huang et al. formulated and validated an oxidative stress-linked prognostic gene signature for LUAD-TCGA, centered around the *MAP3K19* and *NTSR1* genes. Patients with diminished risk scores exhibited a heightened presence of infiltrating immune cells within the tumor microenvironment, correlating with more optimistic immunotherapy outcomes [31].

Necroptosis exerts significant influence on the evolution and metastatic tendencies of LUAD, modulating the inflammatory milieu and the tumor microenvironment [76,77]. In 2022, Zhang et al. isolated 9 povotal necroptosis genes and engineered a risk score via big data. Low-risk patients showcased heightened antitumor immunity and better outcome towards ICIs therapy. In contrast, the high-risk patients exhibited diminished immunotherapeutic outcomes, albeit a positive response to chemotherapy. In addition, they spotlighted *PANX1* gene as a potential target for immunotherapy for the first time, underscoring its significance in immune modulation and prognostic evaluations [32].

Emerging research underscores the involvement of long noncoding RNAs (lncRNAs) in immunological processes, encompassing immune activation, evasion, and cellular infiltration. Similarly, small nucleolar RNAs (snoRNAs) modulate multifaceted gene expression dynamics, influencing chromatin architecture, RNA editing, and translational mechanisms [33,78]. In 2021, Liu et al. introduced a novel risk score model, anchored on six immune-affiliated lncRNA pairs (IRLPs) from 464 LUAD specimens, registering AUC of 0.769. The low-risk group evidenced elevated higher ICI expression and seemingly extracted enhanced benefits from ICIs (*P* < 0.001) [33]. That same year, Wan embarked on an exploration of snoRNA expression patterns across 479 LUAD cases, resulting in the formulation of a tumor-infiltrating immune-linked snoRNA (TIISR) signature. A subdued risk score corresponded to pronounced antitumor immunity, and this scoring system adeptly foretold ICI responses in NSCLC, reflecting in a 3-year AUC of 0.83 and a 5-year AUC of 0.82 [34].

### 2.10 Radiomics-based models

Harnessing artificial intelligence, subtle variances concealed within computed tomography (CT) images can be automatically discerned. Notably, deep learning paradigms exhibit profound potential in augmenting adjuvant NSCLC therapy [79,80]. Immunotherapy for tumors, especially ICIs, has distinct response patterns that often elude quantification by conventional RECIST 1.1 standards. To accommodate clinical research needs, specialized immune-centric response metrics, exemplified by iRECIST (immunotherapy RECIST), have been designed. These allow therapeutic interventions to persist even in the face of radiographic progression [81].

A 2022 multicenter retrospective study introduced and validated the EfficientNetV2-anchored survival benefit prognosis (ESBP) system (Fig. 1D). Drawing from clinically sourced CT scans, the ESBP score gauges the survival advantage of EGFR tyrosine kinase inhibitors and ICIs for stage IV NSCLC patients. A superior ESBP score, exceeding 0.2, correlates with an optimistic prognosis for PFS (HR: 0.36, 95 % CI: 0.19–0.68, *P* < 0.0001). Touted as a non-invasive approach, the ESBP system can bolster diagnostic precision and elevate assessments of survival benefits among radiologists and oncologists [39].

Positron emission tomography (PET)/CT imaging serves as a cornerstone for staging in advanced NSCLC patients. In 2020, Mu et al. harnessed radiomics attributes from PET/CT scans of 99 patients diagnosed with stage IIIB-IV NSCLC, leading to the derivation of a multiparametric radiomics signature (mpRS) adept at forecasting durable clinical benefit. Impressively, the AUC for mpRS spanned 0.86 in the training set, 0.83 in the retrospective validation cohort, and 0.81 in the prospective validation cohort, signaling its commendable precision [42]. In 2021, Mu examined baseline PET/CT images from 210 NSCLC patients undergoing immunotherapy. The research unfolded radiomics signatures tailored to prognosticate cachexia, which in turn held potential to predict durable clinical benefit, PFS, and OS. Through a radiomics lens, it was discerned that patients with an elevated cachexia likelihood experienced truncated PFS and OS, a trend that was particularly pronounced among PD-L1-positive individuals (*P* < 0.05) [43].

In 2022, Humbert et al. intended to unravel the predictive and prognostic ramifications of pathological organ ^18^FDG uptake, linked with organ inflammation, in relation to ICIs treatments for metastatic NSCLC patients. Immuno-induced gastritis was identified as a unique imaging biomarker of better OS. This marker was discernible through early interim ^18^FDG PET scans in roughly 20 % of patients, boasting a 2-year AUC of 0.727 in testing and 0.707 in validation [41]. In 2023, Ventura et al. retrospectively analyzed 44 advanced NSCLC patients and evaluated the predictive and prognostic potency of baseline ^18^F -FDG-PET-CT (PET-CT) radiomic features. Notably, they considered parameters such as PET-Skewness (with an AUC of 0.69) and PET-Median (boasting an AUC of 0.75) [40].

## 3 Conclusions

The ascent of immunotherapy has profoundly transformed NSCLC treatment paradigms. Yet, only a subset of patients manifest tangible responsiveness. Given the fluid dynamics of tumor and TME immunogenicity, placing unwavering faith in a solitary biomarker to anticipate ICI treatment outcomes remains a precarious proposition. Numerous investigations have forged ICI-centric prognostic blueprints for NSCLC, spanning domains such as clinical practice, pathomics, genomics, and radiomics. Anagnostou estimated cTMB, adjusted for tumor purity, which offered a more nuanced prediction of ICB responses. This was achieved by analyzing both whole exome and targeted sequence data across 5449 tumor samples. Subsequently, they pinpointed and validated a refined predictor for immunotherapy responsiveness, encompassing elements like cTMB, receptor tyrosine kinase gene mutations, smoking-related mutational signature, and HLA status. The integration of LIPI with immune-related signatures, such as IeffS-LIPI, appears to offer superior predictive efficacy. Leveraging deep learning methodologies on pre-treatment CT imagery, Deng pioneered and validated a non-invasive, clinically viable model (ESBP) to project additional survival benefits. This underscores the potential of AI networks in aiding oncologists and radiologists to more precisely assess survival advantages. However, it's pertinent to note that many of these findings stem from clinical trials or retrospective evaluations. Consequently, comprehensive prospective studies in real-world setting, enriched by ample sample sizes, are imperative to robustly ascertain the model's applicability and efficacy.

Previous research paid attention to sensitive markers to identify patients more likely benefit from immunotherapy. Recently, increasing trials were developed to investigate ways to enhance the efficacy of ICIs. ABC transporters regulate tumor immune microenvironment by transporting various cytokines to improve sensitivity to anticancer drugs [82]. Activated neoantigen-reactive T cells (NRT) have the ability to resist the growth of tumors expressing specific neoantigens. Immunotherapy based on NRT cells has made achievements in lung cancer [83]. Nanoparticle-based approaches explored new directions and strategies for tumor immunotherapy [84]. In particular, magnetic nanoparticles are a promising option for comprehensively regulating the immune system [85]. The potential application of organoid development in drug efficacy studies for lung cancer was also underscored [86]. Further in depth research on predictive models of immunotherapy should take into account these emerging points to help improve the predictive ability. We can envisage the genesis of multidimensional, multivariable predictive models, sculpted through the synergy of artificial intelligence and expansive big data research. By harnessing multi-platform dynamic assessments—both pre- and post-treatment—and sifting through vast sample sizes, we can hone predictive models to pinnacle prognostic performance. This would, in turn, adeptly identify ideal candidates for immunotherapy, thereby amplifying response rates.

## Data availability statement

The authors confirm that the data supporting the findings of this study are available within the article.

## Funding

This study was supported by 10.13039/501100001809National Natural Science Foundation of China (81472782), the National Key Research and Development Program: The key technology of palliative care and nursing for cancer patients (2017YFC1309201), and Research Fund of Yili Institute of Clinical Medicine (yl2021ms02).

## CRediT authorship contribution statement

**Siqi Ni:** Writing – review & editing, Writing – original draft, Validation, Resources, Investigation, Formal analysis, Data curation, Conceptualization. **Qi Liang:** Writing – review & editing, Writing – original draft, Validation, Resources, Formal analysis, Data curation, Conceptualization. **Xingyu Jiang:** Writing – review & editing, Writing – original draft, Validation, Investigation, Data curation, Conceptualization. **Yinping Ge:** Writing – review & editing, Writing – original draft, Validation, Methodology, Formal analysis, Data curation. **Yali Jiang:** Writing – review & editing, Validation, Resources, Formal analysis, Data curation, Conceptualization. **Lingxiang Liu:** Writing – review & editing, Writing – original draft, Validation, Resources, Methodology, Funding acquisition, Data curation.

## Declaration of competing interest

The authors declare that they have no known competing financial interests or personal relationships that could have appeared to influence the work reported in this paper.

## Abbreviation

ICI: immune checkpoint inhibitors
LIPI: Lung Immune Prognostic Index
NLR: neutrophil-to-lymphocyte ratio
LDH: lactate dehydrogenase
ECOG: Eastern Cooperative Oncology Group
ALB: albumin
ALC: absolute lymphocyte count
RCC: renal cell carcinoma
TIL: tumor-infiltrating lymphocyte
APM: antigen processing machinery
MCS: mutation composite scores
HLA: human leukocyte antigen
